# Characterization of the Active Ingredient and Prediction of the Potential Mechanism of Dahuoluo Pill via Mass Spectrometry with the Network Pharmacology Method

**DOI:** 10.1155/2023/8819534

**Published:** 2023-11-07

**Authors:** Haoran Xu, Yuelin Bi, Xin Feng, Jiaqi Wang, Gengyuan Yu, Tonghua Zhang, Tianyi Li, Xuhua Gao, Runhua Liu, Yu Sun, Hao Wu, Linlin Fang, Chenning Zhang, Yikun Sun

**Affiliations:** ^1^School of Chinese Materia Medica, Beijing University of Chinese Medicine, Beijing 102488, China; ^2^College of Pharmacy, Dalian Medical University, Dalian, Liaoning 116044, China; ^3^Department of Pharmacy, Xiangyang No. 1 People's Hospital, Hubei University of Medicine, Xiangyang 441100, China

## Abstract

The Dahuoluo pill (DHLP) is a classic Chinese patent medicine used to treat rheumatoid arthritis and other conditions. However, there has been no research on the chemical components of DHLP and the mechanisms by which it ameliorates rheumatoid arthritis. Hence, we analysed the chemical components of DHLP and the DHLP components absorbed in blood by using ultraperformance liquid chromatography-Q-exactive-orbitrap-mass spectrometry. We then used network pharmacology to predict the underlying mechanisms by which DHLP ameliorates rheumatoid arthritis. We identified 153 chemical compounds from DHLP, together with 27 prototype components absorbed in blood. We selected 48 of these compounds as potential active ingredients to explore the mechanism. These compounds are related to 88 significant pathways, which are linked to 18 core targets. This study preliminarily reveals the potential mechanisms by which DHLP ameliorates rheumatoid arthritis and provides a basis for further evaluation of the drug's efficacy.

## 1. Introduction

Rheumatoid arthritis (RA) is a chronic and systemic inflammatory disease with a prevalence ranging from 0.4% to 1.3% of the population, depending on sex (women are affected 2–3 times more often than men) and age (the frequency of new RA diagnoses peaks in the sixth decade of life) [1]. The cause of RA is still unknown. The main symptoms of this disease are pain and swelling in the joints of the hands or feet, which interfere with quality of life. These clinical manifestations of RA are stimulated by receptor activators of nuclear factor *κ*B ligand (RANKL), tumour necrosis factor (TNF), and interleukin (IL)-6 [2]. Many drugs have been used to treat RA, including nonsteroidal anti-inflammatory drugs (NSAIDs), glucocorticoids, and disease-modifying antirheumatic drugs (DMARDs) [3]. In recent years, the use of traditional Chinese medicine (TCM) to treat RA has been effective and very popular with patients. TCM can avoid some adverse drug reactions, including infections, nausea, and vomiting, and is more suitable for long-term use [4].

The Dahuoluo pill (DHLP) is made from 50 ingredients, including *Cinnamomum cassia*, *Ephedra*, *Rhizoma coptidis,* and *Angelica sinensis*, which are collected in the *Medicine Standards of the Ministry of Health of People's Republic of China Traditional Chinese Medicine Prescriptions* (Volume 17). DHLP is widely used clinically for its effects of dispelling wind and dampness and relaxing muscles and collaterals, among others. As a famous Chinese patent medicine, the clinical application of DHLP is the treatment of cerebral infarction [5], frozen shoulder [6], and RA [7]. Modern experiments have proved that DHLP can promote peripheral vascular microcirculation, relax blood vessels, and relieve thrombosis [8]. The ingredients in DHLP, such as chlorogenic acid, ligustride I, and Z-ligustilide, have proven anti-inflammatory effects [9]. However, the mechanism by which DHLP ameliorates RA is not completely clear, and further exploration is required.

In recent years, network pharmacology has been widely used in TCM. The disease-gene-target-drug network is a common approach of network pharmacology, and the characteristics of integrity and comprehensiveness of network pharmacology are compatible with the feature of complex components, numerous targets, and diverse regulatory mechanisms in TCM. Therefore, we selected the main chemical components from DHLP as the active ingredients and employed network pharmacology to explore the mechanism by which DHLP ameliorates RA. In the current study, we used ultraperformance liquid chromatography-Q-exactive-orbitrap-mass spectrometry (UPLC-Q-exactive-orbitrap-MS) to identify the chemical components *in vitro* and the components from DHLP absorbed in rat plasma. Furthermore, we screened the main identified components to explore the target and mechanism of DHLP in the treatment of RA through network pharmacology. This experiment provides a new method for the quality control of DHLP and a theoretical basis for its development and utilisation.

## 2. Materials and Methods

### 2.1. Materials

Acetonitrile (MS grade) was supplied by China Thermo Fisher Scientific Co. Ltd. Formic acid (MS grade) was purchased from US ROE company. DHLP (lot no. 19013262) was obtained from Beijing Tongrentang Pharmaceutical Group Co. Ltd. Water was derived from a Milli-Q Ultrapure water purification system (Millipore, Bedford, MA, USA).

Six male Sprague–Dawley rats (300–320 g) were procured from Si Pei Fu Biotechnology Co. Ltd. (Beijing, China). The rats were divided into an experimental group and a normal group and housed in the experimental animal center of the Beijing University of Chinese Medicine (Beijing, China) at 23 ± 2°C and 60% ± 5% relative humidity, with access to water and a normal diet *ad libitum*. The animal experiment protocol was approved by the Animal Care and Use Committee of the Beijing University of Chinese Medicine.

### 2.2. Analysis of the DHLP Chemical Components *In Vitro*

#### 2.2.1. Preparation of the Sample Solution

A portion of DHLP (3.5 g) was cut into small pieces and dissolved in 35 mL of 75% methanol at a ratio of 1 : 10. The stock solution was extracted by the heating reflux method for 1 h. The decoction liquid was centrifuged for 10 min at 12000 rpm and diluted five times, taking 5 *μ*L of the diluent for MS analysis.

#### 2.2.2. Chromatographic Conditions

An ACQUITY UPLC BEH C18 column (1.7 *µ*m, 2.1 × 150 mm; Waters, Milford, MA, USA) was used, with a column temperature of 40°C, an injection volume of 5 *μ*L, a flow rate of 0.3 mL/min, and a mobile phase containing 0.1% formic acid aqueous solution (A) and acetonitrile (B). The gradient elution program was as follows: 0–2 min, 5% B; 2–17 min, 5%–98% B; 17–19 min, 98% B; 19–23 min, 98%–5% B; and 23–25 min, 5% B.

#### 2.2.3. MS Conditions

Electrospray ionisation (ESI) was used for the ion source, positive and negative ions were alternately scanned, the scan mode was a full scan/data-dependent two-stage scan (full scan/ddMS^2^), the scan range was 100–1300 Da, and the capillary temperature was 350°C. The spray voltage in the positive mode was 3200 V, the spray voltage in the negative mode was 3800 V, the sheath gas was 35 arb, and the auxiliary gas was 15 arb. MS^2^ uses three collision energies, low, medium, and high, to perform the second level of the precursor ion. The positive ion mode was 30, 40, and 50 V, and the negative ion mode was 30, 40, and 50 V. The resolution of the primary mass spectra was full scan 70000 full width at half maximum (FWHM), and the resolution of the secondary mass spectra was MS/MS17500 FWHM.

#### 2.2.4. Compound Identification

The raw data were processed with Thermo Xcalibur Qual Browser 3.0.63, which could detect the mass, retention time, and intensity of the peaks in each chromatogram. The chemical components of DHLP were determined by comparison to the relevant data.

### 2.3. Analysis of Absorbed Components *In Vivo*

#### 2.3.1. Drug Intervention

The DHLP sample was dissolved in distilled water. The three rats in the experimental group were given the suspension by intragastric administration at a dose of 10.33 g/kg (clinical five-fold measurement) twice a day for 3 days; the three rats in the normal group were given normal saline via intragastric administration twice a day for 3 days. After the last administration, 0.2 mL of blood was collected in a heparinised microcentrifuge tube from the tail vein at the following time points: 15 min, 30 min, 1 h, 2 h, 4 h, and 6 h. The samples were then centrifuged at 4000 rpm for 10 min, and the supernatant was frozen at −80°C until analysis.

#### 2.3.2. Preparation of Plasma Samples

Plasma samples frozen at −80°C were thawed at room temperature. The plasma samples from different time points were mixed in equal amounts. Then, 360 *µ*L of methanol solution was added to 120 *µ*L of the mixed plasma sample, and the resulting sample was vortexed for 3 min, sonicated in an ice-filled ultrasonic water bath for 10 min, and centrifuged at 4000 rpm for 10 min. The supernatant was removed and blow-dried at 40°C under nitrogen. The residue was dissolved in 100 *µ*L of methanol, vortexed for 30 s, and centrifuged at 12000 rpm for 10 min. The injection volume was 5 *µ*L.

#### 2.3.3. Detection and Analysis

The samples were injected according to the above detection conditions. Qualitative analysis of the prototype composition in the plasma sample was based on the DHLP chemical composition identification results, combined with the retention time and fragment ion information.

### 2.4. Network Pharmacology

#### 2.4.1. Screening of Active Ingredients

The identified compounds from the DHLP chromatogram with an m/z intensity of >10^8^ kg/C were screened as the active ingredients. Meanwhile, to avoid ignoring highly recognised active ingredients, the Chinese Pharmacopoeia (2020 edition) and the relevant literature were used to obtain the ingredients. These findings were merged with the screening results from the ion current diagram. The SwissTargetPrediction website (https://www.swisstargetprediction.ch/) was used for target prediction.

#### 2.4.2. Target Prediction of RA

The human Online Mendelian Inheritance in Man (OMIM, https://www.omim.org/) knowledge base and a human gene database (GeneCards, https://www.genecards.org/) were used to acquire disease targets by entering the keyword “rheumatoid arthritis.”

#### 2.4.3. Construction of the Protein-Protein Interaction (PPI) Network

A bioinformatics website (https://www.bioinformatics.com.cn/) was used to visualise the intersection between the component targets and the disease targets with a Venn diagram. The common targets were imported into the STRING website (https://string-db.org/) to draw the PPI network, and then, Cytoscape 3.7.1 was used to visualise the protein network structure and to analyse the topological characteristics. The medians of the three parameters of degree centrality (DC), betweenness centrality (BC), and closeness centrality (CC) were used as the screening condition. The core targets for the component treatment of RA were obtained by screening twice.

#### 2.4.4. Gene Ontology (GO) Analysis and Kyoto Encyclopedia of Genes and Genomes (KEGG) Enrichment Analysis

The direct targets of the ingredients on RA were imported into the DAVID website (https://david.ncifcrf.gov/). GO and KEGG enrichment maps were made through a bioinformatics website (https://www.bioinformatics.com.cn/).

#### 2.4.5. Network Construction

The TCM, components, targets, and pathways of DHLP were imported into Cytoscape 3.7.1 to establish a TCM-component-target-pathway network.

## 3. Results

### 3.1. Identification of the Chemical Constituents

**Figure 1:**
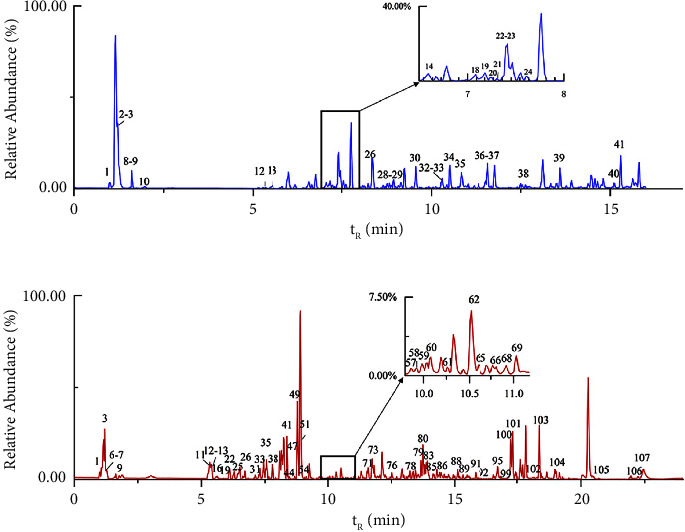
Base peak ion flow diagram of DHLP under negative ion (a) and positive ion (b) by UPLC-Q-exactive-orbitrap-MS.

**Figure 2:**
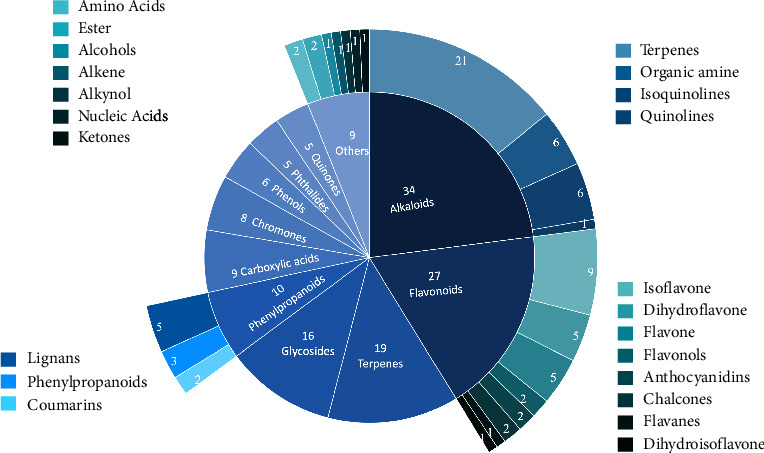
Compounds classified.

Based on the optimised chromatographic and MS analysis conditions, we analysed the components of DHLP. We identified that 153 compounds were under the positive and negative ion modes, including 34 alkaloids, 27 flavonoids, 19 terpenes, 16 glycosides, and 10 phenylpropanoids. The base peak integration is shown in Figure 1. The classification diagrams of the identified compounds are shown in Figure 2. The chemical composition lists are shown in Table 1 for the negative ion mode and Table 2 for the positive ion mode.

#### 3.1.1. Alkaloids

**Figure 3:**
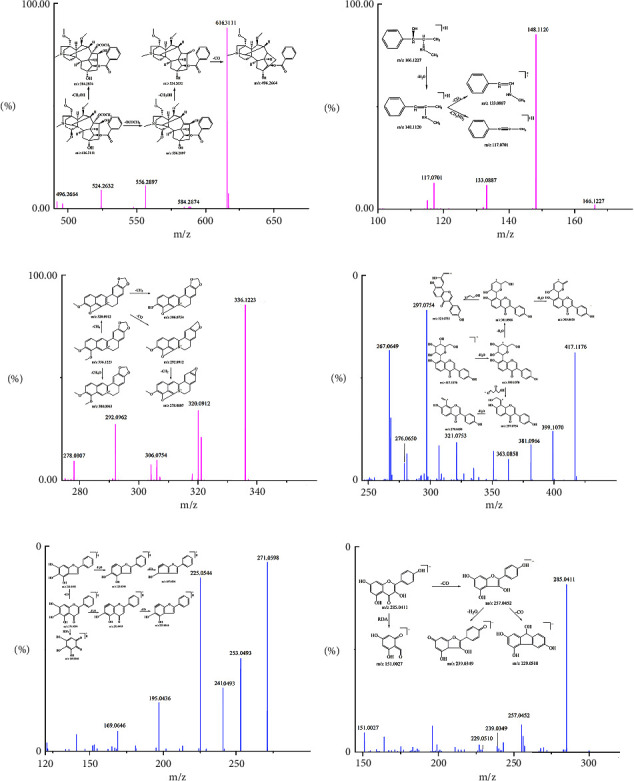
The MS/MS spectrums of alkaloids and flavonoids in positive ion mode or negative ion mode and their probable fragmentation pathway: (a) hypaconitine, (b) ephedrine, (c) berberine, (d) puerarin, (e) baicalin, and (f) kaempferol.

Hypaconitine is a terpene alkaloid, and the diagnostic ions related to it include [M + H − CH_3_OH]^+^, [M + H − OCOCH_3_]^+^, and [M + H − OCOCH_3_ − CH_3_OH]^+^. In the positive ion flow diagram, the retention time of peak 67 is 10.25 min, and its quasi-molecular ion peak is m/z 616.3111 [M + H]^+^. The fragment ions coincide with that of hypaconitine, so we determined that the compound is hypaconitine. The cleavage law of hypaconitine and the secondary mass spectrum are shown in Figure 3(a).

Ephedrine is an organic amine alkaloid. The N atom in its side chain is relatively active, and the substituents attached to the N atom are easily lost by MS collisions. In the positive ion flow diagram, the retention time of peak 11 is 5.38 min, and its quasi-molecular ion peak is m/z 166.1227 [M + H]^+^. At the same time, the characteristic fragments of m/z 148.1120 [M + H − H_2_O]^+^, [M + H − H_2_O − CH_3_]^+^, and [M + H − H_2_O − NH_2_CH_3_]^+^ can be observed. Hence, we determined that the compound is ephedrine. The cleavage law of ephedrine and the secondary mass spectrum are shown in Figure 3(b).

Berberine is an isoquinoline alkaloid. It is prone to [M + H − CH_3_]^+^, and then, further removal of the H connected to N produces [M + H − CH_4_]^+^, which can remove CO at the same time to get [M + H − CH_4_ − CO]^+^ and can also remove CH_2_ to get [M + H − CH_4_ − CH_2_]^+^. The quasi-molecular ion peak and the fragment ions of compound 54 coincide with berberine. The cleavage law of berberine and the secondary mass spectrum are shown on the left side of Figure 3(c).

#### 3.1.2. Flavonoids

Puerarin is an isoflavone, which mainly undergoes cleavage of the D ring-glycosidic bond, forming a stable conjugate between the A, B, and C rings, which is not easy to break. The pyrolysis characteristics of puerarin are m/z 399, 381, 363, 321, 297, and 279, which coincide with compound 22. The cleavage law of puerarin and the secondary mass spectrum are shown on the right side of Figure 3(d).

Baicalein is an isoflavone, and its cleavage characteristics are similar to that of puerarin. In the positive ion flow diagram, compound 59 coincides with baicalein, whose fragment ions include m/z 253.0493 [M + H − H_2_O]^+^, 241.0493 [M + H − CO]^+^, 225.0544 [M + H − H_2_O − CO]^+^, and 195.0436 [M + H − 2CO − H_2_O]^+^. At the same time, the compound can undergo an RDA reaction to generate the fragment ion m/z 169.0646. The cleavage law of baicalein and the secondary mass spectrum are shown on the left side of Figure 3(e).

Kaempferol is a flavonol, which loses CO through the cleavage of the C ring to produce m/z 257.0452 [M − H − CO]^−^ (Table 3). Further loss of CO or H_2_O produces m/z 229.0510 [M − H − 2CO]^−^ or 239.0349 [M − H − CO − H_2_O]^−^, respectively. It also can undergo an RDA reaction to generate fragment ion m/z 151.0027. The cleavage law of kaempferol and the secondary mass spectrum are shown on the right side of Figure 3(f).

### 3.2. Network Pharmacology

#### 3.2.1. Chemical Composition Screening and Target Prediction

We selected a total of 48 compounds: 36 of the identified compounds with the intensity >10^8^ from the DHLP chromatogram and 12 compounds from the Chinese Pharmacopoeia (2020 edition) and the related literature. We used the SwissTargetPrediction website (https://www.swisstargetprediction.ch/) to predict targets, thereby obtaining a total of 1480 targets.

#### 3.2.2. Target Prediction for RA

**Figure 4:**
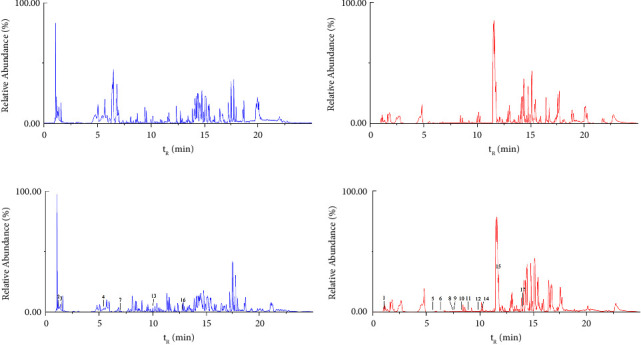
BPI of blank plasma in negative ion (a) and positive ion (b) and plasma samples after administration of DHLP in negative ion (c) and positive ion (d) by UPLC-Q-exactive-orbitrap-MS.

We found 1196 disease targets after searching the OMIM and GeneCards databases with “rheumatoid arthritis” as a keyword, and then screening, merging, and removing duplicates (Figure 4).

#### 3.2.3. Construction of the PPI Network

**Figure 5:**
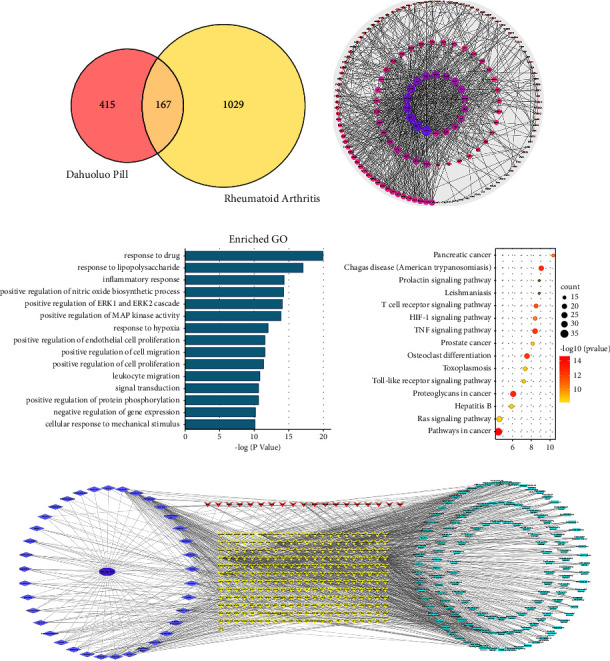
The Venn diagram of common targets from the components and disease (a) and the PPI network of 167 targets in DHLP (b). The GO enrichment analysis (c) and the KEGG pathways analysis (d) of 167 targets. Traditional Chinese medicine-component-target-pathway network (e).

We generated the Venn diagram through the bioinformatics website (Figure 5(a)). There are 167 common targets.

The STRING website is a functional protein association network that can predict PPIs. After importing all the targets into the website, we obtained and saved the PPI network in TSV format. We used Cytoscape 3.7.1 to analyse the topological characteristics of the protein network structure and to screen the core targets of the components for the treatment of diseases, as shown in Figure 5(b) and Table 4. The middle and innermost targets in Figure 5(b) are the results after the first screening, while the innermost layer is the result of the second screening. This later shows the core targets, sorted by the degree value from deep to shallow and from large to small. After sorting the 18 core targets, we found that AKT1 (degree = 52), MAPK1 (degree = 50), STAT3 (degree = 50), VEGFA (degree = 48), MAPK8 (degree = 45), EGFR (degree = 44), and TNF (degree = 43) are the key targets in this network.

#### 3.2.4. GO Analysis and KEGG Enrichment Analysis

With a false discovery rate (FDR) <0.001 as the screening condition, we obtained 82 GO biological items, including response to the drug, response to lipopolysaccharide, the inflammatory response, positive regulation of nitric oxide, biosynthetic process, and positive regulation of ERK1 and ERK2 cascade. The 15 biological processes ranked according to the FDR value are shown in Figure 5(c). We speculate that DHLP may ameliorate RA via complex multichannel synergy.

To further explore the mechanism of action of DHLP in treating RA, we conducted KEGG enrichment analysis on 167 targets and screened out 88 related pathways according to FDR <0.01. After sorting according to the FDR value, the top 15 ranked pathways are shown in Figure 5(d). They include the TNF signalling pathway, hepatitis B, pathways in cancer, Chagas disease (American trypanosomiasis), the toll-like receptor signalling pathway, and other related pathways.

#### 3.2.5. Construction of the TCM-Component-Target-Pathway Network

To clarify the relationship between medicinal materials, ingredients, targets, and pathways, we used Cytoscape 3.7.1 to construct a TCM-component-target-pathway network (Figure 5(e)). Through this network, we can visually demonstrate the effective substances in DHLP that treat RA, as well as their possible mechanisms of action.

## 4. Discussion

DHLP is a famous TCM that contains a wide variety of medicinal materials. However, DHLP is a complex mixture of a wide variety of herbal medicines, and this composition may cause problems such as difficult drug quality control and the restriction of medicines [38, 39]. UPLC-Q-exactive-orbitrap-MS has high sensitivity and selectivity and is widely used in the analysis of chemical components in complex chemical systems of TCM. Therefore, we used UPLC-Q-exactive-orbitrap-MS in the positive and negative ion modes to quickly identify the chemical components in DHLP and the DHLP components absorbed in blood. According to the retention time, molecular ion peaks, and fragments, as well as the chemical composition of DHLP based on the related literature, we identified 117 compounds in the positive ion mode and 41 compounds in the negative ion mode, including 34 alkaloids, 27 flavonoids, 19 terpenes, 16 glycosides, and 10 phenylpropanoids. Furthermore, we identified 27 prototype components absorbed in blood based on comparison with the *in vitro* ingredients, including 11 flavonoids, 3 glycosides, and 2 alkaloids. This information provides an experimental basis to find new active ingredients of DHLP, to improve quality control standards, and to guide the rational clinical use of drugs.

Network pharmacology is based on biological networks that reveal the interconnections between complex diseases, symptoms, and prescriptions. Many scholars have used this approach to conduct in-depth research on TCM. Based on network pharmacology, we identified 48 compounds, 18 core targets, and 88 related pathways in DHLP that may be related to the treatment of RA. Among them, the top seven core targets are AKT1, MAPK1, STAT3, VEGFA, MAPK8, EGFR, and TNF. MAPK1 is also called ERK2, and a high concentration of epidermal growth factor (EDF) promotes the expression of COX-2 by stimulating the activity of ERK1/2-MAPK to participate in the inflammatory response of RA [40]. TNF participates in the formation of pannus and can promote cartilage destruction and aggravate inflammation to promote the formation and development of RA [41]. Therefore, the chemical components in DHLP may treat RA by regulating related proteins such as MAPK1 and TNF. KEGG enrichment analysis revealed enrichment of osteoclast differentiation and TNF, toll-like receptor, and HIF-1 signalling pathways, among others. Mesenchymal stem cells can directly inhibit osteoclast differentiation by producing NF-*κ*B receptor activator ligands to induce osteoprotegerin production [42]. The toll-like receptor signalling pathway plays an important role in joint destruction caused by chronic expression of proinflammatory cytokines and chemokines [43]. The HIF-1 signalling pathway is an important angiogenesis pathway in RA. HIF-1 affects cell reactivity in synovial tissue under hypoxia to promote the infiltration of macrophages and other inflammatory cells, producing vascular endothelial growth factor (VEGF) and increasing the release of TNF inflammatory mediators, which leads to the persistence of synovitis [44]. Therefore, we speculate that DHLP may ameliorate RA by regulating osteoclast differentiation, by altering the toll-like receptor and HIF-1 signalling pathways, and by acting through other mechanisms.

The prototype components absorbed in blood include glycosmisic acid, costunolide, wogonoside, and chlorogenic acid. Chrysophanic acid could alleviate the pathological changes of osteonecrosis of the femoral head by augmenting osteogenesis and retarding adipogenesis in the scenario of ethanol administration, which may appear during the course of RA [45]. We identified glycosmisic acid, which is found in *Glycyrrhiza uralensis*, as a prototype component and an active ingredient. It exerts anti-inflammatory effects by inhibiting the expression of NF-*κ*B, IL-6, and IL-8 [32]. Wogonoside, which is mainly contained in *Radix scutellariae*, attenuates IL-1*β*-induced extracellular matrix degradation and hypertrophy in mouse chondrocytes by suppressing activation of NF-*κ*B/HIF-2*α* in the PI3K/AKT pathway [33] and can also suppress lipopolysaccharide-stimulated production of inflammatory factors by repressing the activation of the JNK/c-Jun signalling pathway in macrophages [36]. The absorbed components mainly ameliorate RA by acting on targets such as STAT3, IL-2, and MMP12 through the toll-like receptor, MAPK, T-cell receptor, and other pathways.

In summary, we employed UPLC-Q-extactive-orbitrap-MS and identified 153 chemical compounds from DHLP and 27 prototype components of DHLP absorbed in blood, and we explored the potential mechanism for the treatment of RA through the method of network pharmacology. We screened 48 potential active ingredients based on the MS results and the related literature. We identified 1480 potential targets based on these 48 compounds, with 1196 RA-related disease targets, including 167 common targets for DHLP and RA. The GO biological process and KEGG signalling pathway enrichment analysis for these targets predicted that DHLP could regulate MAPK1, STAT3, AKT1, MAPK8, TNF, and other targets and that DHLP could regulate TNF, toll-like receptor, HIF-1, and other signalling pathways as well as osteoclast differentiation to suppress inflammation and to regulate immune function to treat RA. The results of the current study provide important experimental data and bioinformatics analysis for further development and rational use of DHLP.

## Figures and Tables

**Table 1:** The identified chemical constituents in DHLP by UPLC-Q-exactive-orbitrap-MS under negative ion.

No.	*t *_*R*_ (min)	Measured value	MS/MS	Formula	Identification	Error (ppm)	Source
1	1.08	319.1520	167.0809; 139.0866; 137.0708; 123.0555; 109.0763	C_17_H_18_O_6_	Evofolin B [10]	1.88	Cinnamomum cassia
2	1.21	493.1222	331.1624; 313.0565; 169.0134; 151.0029; 125.0234	C_19_H_26_O_15_	Gallic acid-O-diglucoside [11]	−4.66	Rhubarb
3	1.22	191.0555	129.0181; 111.0077	C_6_H_8_O_7_	Citric acid [12]	2.09	Radix polygoni multiflora/radix rehmanniae preparata
4	1.24	331.0679	211.0244; 169.0136; 125.0234	C_13_H_16_O_10_	Glucogallin [13]	−2.42	Radix aconiti kusnezoffii
5	1.29	169.0135	125.0234; 150.9698	C_7_H_6_O_5_	Gallic acid [12]	4.14	Radix polygoni multiflori
6	1.41	341.1083	179.0556; 135.0452	C_15_H_18_O_9_	1-O-Caffeoyl glucoside [14]	−0.88	Rhizoma drynariae
7	1.49	191.0555	173.0083; 127.0386; 111.0076	C_7_H_12_O_6_	Quinic acid [13]	3.14	Radix aconiti kusnezoffii
8	1.63	243.0634	111.0188; 68.0129	C_9_H_12_N_2_O_6_	Uridine [15]	−4.94	Radix rehmanniae preparata
9	1.64	331.0679	271.0461; 169.0135; 125.0234	C_13_H_16_O_10_	Gallic acid-O-glucoside [11]	−2.42	Rhubarb
10	1.87	169.0135	125.0234	C_7_H_6_O_5_	Gallic acid [13]	4.14	Radix aconiti kusnezoffii
11	3.85	153.0184	109.0284; 91.0177; 81.0333	C_7_H_6_O_4_	Protocatechin [10]	5.88	Cinnamomum cassia
12	5.43	353.0880	191.0555	C_16_H_18_O_9_	Chlorogenic acid [16]	−0.57	Ephedra/rhizoma coptidis/angelica sinensis
13	5.45	431.0991	311.0562; 269.0453; 265.0520; 225.0553	C_21_H_20_O_10_	Anthraglycoside B [10]	−1.62	Cinnamomum cassia
14	6.19	289.1455	245.1552	C_15_H_14_O_6_	Catechin [12]	1.38	Radix polygoni multiflori
15	6.30	207.0299	192.0423; 148.0522	C_11_H_12_O_4_	Trans-3,4-dimethoxycinnamic acid [17]	0	Rhizoma coptidis
16	6.93	300.9990	283.9962; 257.0093	C_14_H_6_O_8_	Ellagic acid [13]	0	Radix aconiti kusnezoffii/radix paeoniae rubra/myrrha
17	6.97	549.1635	255.0663; 135.0078	C_26_H_30_O_13_	Celery glycyrrhizin [18]	−4.01	Glycyrrhiza uralensis
18	7.08	417.1196	255.0663; 135.0078	C_21_H_22_O_9_	Liquiritin [18]	−1.19	Glycyrrhiza uralensis
19	7.14	405.0690	243.0661	C_20_H_22_O_9_	2,3,5,4′-Tetrahydroxystilbene glucoside [12]	0.25	Radix polygoni multiflori
20	7.21	167.0342	123.0076	C_8_H_8_O_4_	Vanillic acid [19]	4.79	Angelica sinensis
21	7.33	193.0500	179.0300; 149.0598	C_10_H_10_O_4_	Ferulic acid [19]	3.11	Angelica sinensis
22	7.39	623.2018	461.1673; 161.0236	C_29_H_36_O_15_	Acteoside [15]	4.65	Radix rehmanniae preparata
23	7.44	579.8508	459.1163; 271.0614; 151.0027	C_27_H_32_O_14_	Naringin [14, 20, 21]	1.55	Qingpi/rhizoma drynariae
24	7.51	295.1198	173.0085	C_13_H_12_O_8_	2-O-Caffeoylmalic acid [16]	−0.34	Ephedra
25	7.73	609.1401	301.0353	C_28_H_34_O_15_	Hesperidin [22]	0.82	Qingpi
26	8.38	631.9897	193.0500; 175.0394	C_31_H_40_O_15_	Rehmaionoside/isomartynoside [15]	−0.46	Radix rehmanniae preparata
27	8.63	253.0506	225.0561	C_15_H_10_O_4_	Chrysophanic acid [23]	0	Rhubarb
28	8.65	447.0940	285.0406	C_22_H_24_O_10_	2,3,5,4′-Tetrahydroxystilbene-2-O-acetyl-glucoside [12]	−0.89	Radix polygoni multiflori
29	8.82	255.0664	153.0185; 135.0185; 119.0492	C_15_H_12_O_4_	Isoliquiritigenin [18]	−1.57	Glycyrrhiza uralensis
30	8.89	285.0411	257.0452; 239.0349; 229.0510; 151.0027	C_15_H_10_O_6_	Kaempferol [24]	−2.11	Glycyrrhiza uralensis/rhizoma drynariae/ephedra
30	9.61	837.3923	661.3602; 485.3271; 351.0572	C_42_H_62_O_17_	Licoricesaponin G2 [18]	−1.07	Glycyrrhiza uralensis
32	10.29	269.0458	241; 0508; 225.0550	C_15_H_10_O_5_	Aloe-emodin [23]	−1.12	Rhubarb
33	10.34	299.0570	284.0327	C_16_H_12_O_6_	Kaempferide [24]	−3.01	Clove
34	10.49	821.3983	645.3605; 469.3316; 351.0573; 193.0349; 113.0233	C_42_H_62_O_16_	Glycyrrhizic acid [18]	−2.19	Glycyrrhiza uralensis
35	10.96	807.4189	351.0571; 193.0350.113.0233	C_42_H_64_O_15_	Glycyrrhizin B2 [18]	−2.11	Glycyrrhiza uralensis
36	11.54	283.0255	239.0349; 183.0445	C_15_H_8_O_6_	Rheinic acid [23]	−2.47	Rhubarb
37	11.62	327.1241	177.0915; 165.0549; 147.0802	C_15_H_20_O_8_	Dihydromelilotoside [10]	2.14	Cinnamomum cassia
38	12.53	371.1506	193.0867; 177.0192; 163.0400; 123.0441	C_20_H_20_O_7_	Glycosmisic acid [10]	−2.42	Cinnamomum cassia
39	13.50	351.0878	307.0975; 237.0918	C_20_H_32_O_5_	Cinncassiol D4 [10]	−1.14	Cinnamomum cassia
40	15.18	435.1436	417.133	C_21_H_22_O_10_	Dihydro kaempferol-7-O-rhamnoside [14]	−3.68	Rhizoma drynariae
41	15.34	283.0614	240.0427	C_16_H_12_O_5_	Emodin monomethyl ether [23]	−0.71	Rhubarb

**Table 2:** The identified chemical constituents in DHLP by UPLC-Q-exactive-orbitrap-MS under positive ion.

No.	*t*_*R*_ (min)	Measured value	MS/MS	Formula	Identification	Error (ppm)	Source
1	1.07	175.1188	158.0922; 130.0974; 116.0704; 70.0657	C_6_H_14_N_4_O_2_	Arginine [24]	0.57	Rhizoma arisaematis
2	1.14	295.0961	277.1029; 259.0919; 241.0819; 211.0710	C_18_H_14_O_4_	Isoimperatorin [25]	1.36	Saposhnikovia divaricata
3	1.15	127.0389	109.0285	C_6_H_6_O_3_	5-Hydroxymethyl-2-furaldehyde [19]	1.57	Angelica sinensis
4	1.16	145.0494	127.0389; 109.0287	C_6_H_8_O_4_	Pyranone [26]	1.38	Glycyrrhiza uralensis
5	1.22	305.1015	287.1230; 273.1275	C_16_H_16_O_6_	Hesperetin dihydrochalcone [24]	1.64	Clove
6	1.23	280.1390	262.1281	C_11_H_21_NO_7_	N-Fructosyl valine [24]	0.36	Rhizoma arisaematis
7	1.24	256.0724	147.0442	C_15_H_12_O_4_	Liquiritigenin [16]	4.69	Glycyrrhiza uralensis
8	1.28	230.1531	166.0857; 145.0491; 128.1065	C_15_H_19_ON	Atractylenolactam [22]	3.48	Atractylodes macrocephala koidz
9	1.82	121.0648	105.0449; 93.0702	C_8_H_8_O	1-Acetophenone [19]	0	Angelica sinensis
10	3.62	394.2514	376.2479; 358.2379	C_22_H_35_NO_5_	Karakolidine [13]	−1.01	Radix aconiti kusnezoffii
11	5.38	166.1262	148.1120; 133.0887; 117.0701	C_10_H_15_NO	Ephedrine/pseudoephedrine [26]	1.20	Ephedra
12	5.38	148.1120	133.0887	C_10_H_13_N	5,6,7,8-Tetrahydro-4-methylquinoline [26]	0.68	Ephedra
13	5.48	408.2646	390.2631	C_23_H_37_NO_5_	Isotalatizidine [13]	4.90	Radix aconiti kusnezoffii
14	5.49	378.2634	360.2529	C_22_H_35_NO_4_	Carmichaeline [27]	1.32	Aconitum carmichaelii debx
15	5.59	355.2476	337.0915; 319.0813; 301.0712	C_20_H_34_O_5_	Incensole trioxide [19]	0.84	Frankincense
16	5.62	360.2437	342.2431	C_22_H_33_NO_3_	Napelline [13]	5.00	Radix aconiti kusnezoffii
17	5.64	180.1382	162.1276; 147.1041; 132.0813	C_11_H_17_NO	Mephedrone [26]	0.56	Ephedra
18	5.66	408.2745	390.2631	C_23_H_37_NO_5_	Talatizidine [27]	−0.24	Aconitum carmichaelii debx
19	5.73	193.0494	161.0596; 133.0648; 149.0589; 105.0702	C_10_H_8_O_4_	Scopoletin [25]	0.52	Saposhnikovia divaricata
20	5.81	358.2372	340.2267	C_22_H_31_NO_3_	Songorine [13, 25]	1.40	Aconitum carmichaelii debx/radix aconiti kusnezoffii
21	5.95	355.1533	337.0915; 319.0809	C_21_H_22_O_5_	14-Acetyl-kirilightacyl-12-transtractylodes triol [21]	1.97	Atractylodes macrocephala koidz
22	6.06	417.1176	399.1070; 381.0966; 351.0860	C_21_H_20_O_9_	Puerarin [17]	0.96	Radix puerariae
23	6.19	454.2795	436.2694; 404.2429	C_24_H_39_NO_7_	Fuziline [13, 25]	0.88	Aconitum carmichaelii debx/radix aconiti kusnezoffii
24	6.26	447.1281	429.1180; 411.1072; 393.0968	C_22_H_22_O_10_	Oxyayanin b trimethyl ether [17]	1.12	Radix puerariae
25	6.41	438.2542	420.2742; 388.2481	C_24_H_39_NO_6_	Neoline [13, 25]	1.83	Aconitum carmichaelii debx/radix aconiti kusnezoffii
26	6.60	417.1183	255.0649	C_21_H_20_O_9_	Daidzin [17]	−0.72	Radix puerariae
27	6.77	422.2836	390.2633	C_24_H_39_NO_5_	Talatizamine [13]	−3.08	Radix aconiti kusnezoffii
28	6.95	344.2508	326.2475	C_22_H_33_NO_2_	Bullatine A [13]	−0.58	Radix aconiti kusnezoffii
29	7.05	549.1598	531.1501; 513.1383; 495.1281; 411.1072; 393.0967; 375.0859	C_26_H_28_O_13_	Chrysin-6-C-arabinose-8-C-glucoside [28]	0.91	Radix scutellariae
30	7.16	163.0389	135.0441; 117.0337; 105.0704; 89.0390	C_9_H_6_O_3_	Umbelliferone [25]	0.61	Saposhnikovia divaricata
31	7.20	286.1434	269.1169; 239.0707; 237.0909; 209.0961; 194.0724; 143.0491; 115.0544	C_17_H_19_NO_3_	Coclaurine [29]	1.40	Radix linderae
32	7.20	463.0861	287.0546	C_21_H_18_O_12_	5,7-dihydroxyl-2′-methoxyflavone-7-O-Gluconaldehyde [28]	2.16	Radix scutellariae
33	7.20	208.0966	165.0909; 150.0678	C_11_H_13_NO_3_	Thalietrine [17]	0.96	Rhizoma coptidis
34	7.23	245.1170	227.0700; 199.0752; 141.0696; 128.0624; 115.8399; 91.0546	C_15_H_16_O_3_	Linderalactone [29]	0.82	Radix linderae
35	7.27	319.1172	301.1067; 283.0962; 255.1013; 227.1065; 164.0467	C_17_H_18_O_6_	Agarotetrol [30]	1.25	Aquilaria agallocha
36	7.49	581.1868	435.1288; 273.0755; 153.0182	C_27_H_32_O_14_	Naringenin [14]	−0.52	Rhizoma drynariae
37	7.54	453.1750	291.1222; 273.1117; 259.0960; 243.0649; 219.0649; 205.0494	C22H28O10	5-O-Methylvisammioside [31]	1.10	Saposhnikovia divaricata
38	7.57	319.1169	301.1065; 283.0960; 255.1012; 227.1063; 164.0466	C_17_H_18_O_7_	Aquilarone B [30]	2.19	Aquilaria agallocha
39	7.65	317.0653	302.0419; 285.0396	C_16_H_12_O_7_	Rhamnetin/isorhamnetin [24]	0.95	Clove
40	7.80	611.1964	449.1447; 413.1447; 431.1324; 369.0963; 345.0966; 303.0859; 263.0545	C_28_H_34_O_15_	Neohesperidin [32, 33]	0.98	Qingpi
41	7.95	477.1024	301.0703; 286.0468	C_22_H_20_O_12_	5,7,2′-trihydroxy-6-methoxyflavone-7-O-glucuraldehyde glycosides [28]	0.63	Radix scutellariae
42	8.28	419.1335	257.0806; 239.0702; 229.0858	C_21_H_22_O_9_	Liquiritin [26]	0.48	Glycyrrhiza uralensis
43	8.28	419.1720	223.9430; 211.0752	C_22_H_26_O_8_	Flavaspidic acid ab [34]	−4.77	Guanzhong
44	8.31	339.1454	323.1136; 294.1117	C_20_H_20_NO_4_	Jatrorrhizine [17]	3.24	Rhizoma coptidis
45	8.33	590.2904	572.2814; 540.2599; 508.2399	C_31_H_43_NO_10_	14-benzoylmesaconine [13]	−3.90	Radix aconiti kusnezoffii
46	8.37	271.0598	253.0493; 243.0549	C_15_H_10_O_5_	7,8,4′-Trihydroxyisoflavone [17]	1.11	Radix puerariae
47	8.49	447.0930	271.0598; 253.0494	C_21_H_18_O_11_	Baicalin [28]	−1.79	Radix scutellariae
48	8.50	431.1340	269.0805	C_22_H_22_O_9_	Ononin [17]	−0.93	Radix puerariae/Glycyrrhiza uralensis
49	8.51	269.0434	241.0492	C_15_H_8_O_5_	Coumestrol [17]	3.72	Radix puerariae
50	8.69	604.3008	586.3019; 572.2835; 554.2741; 540.2741; 522.2476	C_32_H_45_NO_10_	14-Benzoylaconine [13]	4.96	Radix aconiti kusnezoffii
51	8.69	255.0647	237.0543; 227.0699; 199.0751	C_15_H_10_O_4_	Daidzein [17]	1.96	Radix puerariae
52	8.72	193.1221	175.0753; 147.0441	C_12_H_16_O_2_	4-*n*-Pentylbenzoic acid [24]	1.04	Rhizoma arisaematis
53	8.84	291.1222	273.1118; 259.0953; 243.0649; 219.0649	C_16_H_18_O_5_	5-O-Methylvisamminol [31]	−3.26	Saposhnikovia divaricata
54	8.88	337.1239	321.0967; 320.0914; 292.0970	C_20_H_18_NO_4_	Berberine [17]	1.72	Rhizoma coptidis
55	8.95	461.1071	285.0752; 270.0518	C_22_H_20_O_11_	Wogonoside [28]	1.52	Radix scutellariae
56	8.96	574.2904	542.2746; 510.2484	C_31_H_43_NO_9_	14-Benzoylhypacoine [13]	4.88	Radix aconiti kusnezoffii
57	9.11	572.2804	554.2769; 540.2573; 522.2494	C_31_H_41_NO_9_	Dehydrated benzoylmesacoine [13]	−4.89	Radix aconiti kusnezoffii
58	9.11	572.3168	540.2573; 508.2308	C_32_H_45_NO_8_	14-O-Anisoylneoline [13]	−4.89	Radix aconiti kusnezoffii
59	9.12	203.1268	185.1328; 175.0387; 157.1009	C_10_H_18_O_4_	Sebacic acid [24]	4.92	Rhizoma arisaematis
60	9.13	271.0598	253.0493; 241.0493; 225.0544; 197.0956; 169.0646	C_15_H_10_O_5_	Baicalein [28]	1.11	Radix scutellariae
61	9.35	323.1136	294.0876	C_19_H_16_NO_4_	Berberrubine [17]	4.95	Rhizoma coptidis
62	9.37	588.3069	556.2897; 524.2624	C_32_H_45_NO_9_	14-benzoyl-methoxy-hypaconine [13]	3.40	Radix aconiti kusnezoffii
63	9.88	285.0753	270.0518; 267.0646; 257.0796	C_16_H_12_O_5_	3′-Methoxyldaidzein [17]	1.40	Radix puerariae
64	9.95	327.1222	221.0806; 107.0494	C_19_H_18_O_6_	Qinanone g (isomer) 2 [30]	1.53	Aquilaria agallocha
65	9.97	283.0960	192.0414; 164.0468	C_17_H_14_O_4_	6,8-Dihydroxyl-2-(2-phenethyl) chromone [30]	1.41	Aquilaria agallocha
66	10.10	233.1534	215.1428; 187.1479; 159.1167; 145.1011; 131.0855	C_15_H_20_O_2_	Costunolide [30]	0.86	Radix aucklandiae
67	10.25	616.3111	556.2897; 524.2632; 584.2874; 496.2664	C_33_H_45_NO_10_	Hypaconitine [13]	0.81	Radix aconiti kusnezoffii
68	10.50	471.3462	407.3319; 121.1012	C_30_H_46_O_4_	1-Carbonyl-*β*-boswellic acid [35]	1.49	Frankincense
69	10.68	327.1222	221.0807; 107.0484	C_19_H_18_O_5_	Inanone g (isomer 1) [19]	1.53	Aquilaria agallocha
70	10.69	267.1014	252.0428; 224.0479	C_17_H_14_O_3_	Dracorhodin [36]	4.87	Resina draconis
71	10.72	403.1380	388.1140; 373.0912; 355.0816; 327.0856	C_21_H_22_O_8_	Nobiletin [32, 33]	1.74	Qingpi
72	10.80	217.0493	202.0259; 189.0905; 174.0310; 161.0594; 146.0358	C_12_H_8_O_4_	Xanthotoxin [25]	0.92	Saposhnikovia divaricata
73	10.90	189.0909	161.0958; 147.0440; 130.0776; 119.0492; 105.0337	C_12_H_12_O_2_	Z-Butylidenephthalide [19]	0.53	Angelica sinensis
74	10.94	249.1484	231.1373; 213.1272; 203.9383; 189.1274; 163.0751; 135.0803	C_15_H_20_O_3_	Atractylenolide III [22]	4.42	Atractylodes macrocephala koidz
75	11.00	267.1010	176.0467; 161.0969; 137.0229	C_17_H_14_O_3_	7-Hydroxyl-2-(2-phenethyl) chromone [30]	2.25	Aquilaria agallocha
76	11.00	323.2576	305.2469; 287.2367; 269.2253	C_20_H_34_O_3_	Incensole oxide [19]	1.55	Frankincense
77	11.46	479.1162	317.1378; 299.6608	C_22_H_22_O_12_	Isorhamnetin-3-O-glucoside [24]	4.59	Clove
78	11.52	285.0753	270.0518	C_16_H_12_O_5_	Wogonin [28]	1.40	Radix scutellariae
79	11.77	352.1172	337.0939; 308.0923	C_20_H_17_NO_5_	8-O-Berberine [17]	1.99	Rhizoma coptidis
80	11.80	257.0804	239.0698; 229.2530	C_15_H_12_O_4_	Liquiritigenin [26]	1.17	Glycyrrhiza uralensis
81	11.92	203.0709	174.0316; 145.0287	C_12_H_12_O_3_	3-butenyl-7-hydroxyphthalide [19]	2.46	Angelica sinensis
82	11.95	567.2014	549.1899; 531.1796; 283.0959; 267.1010; 255.1012; 239.1062	C_34_H_30_O_8_	AH13 [30]	−0.18	Aquilaria agallocha
83	12.43	355.1040	191.0337; 161.0598	C_16_H_18_O_9_	Chlorogenic acid [17]	−4.79	Rhizoma coptidis
84	12.63	194.1167	177.0898; 151.0627; 135.0439; 91.0544	C_11_H_15_NO_2_	Racsalsoline [29]	4.64	Radix linderae
85	13.37	475.1534	371.0909; 341.0806	C_31_H_23_O_5_	Nordacorubin [37]	1.26	Resina draconis
86	13.62	233.1537	215.1429; 187.1480; 151.0753	C_15_H_20_O_2_	Atractylodes lactone II [21]	−0.43	Atractylodes macrocephala koidz
87	13.85	189.0909	171.1165; 159.0801; 131.0854	C_12_H_12_O_2_	Butylidenephthalide [19]	0.53	Angelica sinensis
88	13.89	195.0650	178.0774; 148.9767	C_10_H_10_O_4_	Ferulic acid [24]	1.03	Rhizoma arisaematis/clove
89	13.95	225.1118	207.1014; 151.0392	C_12_H_16_O_4_	Chuanxionglide II [19]	1.33	Angelica sinensis
90	13.97	217.1584	157.1010; 147.1165; 143.0854; 131.0853; 129.0699; 121.0648; 119.0856	C_15_H_20_O	Atractylone [21]	1.38	Atractylodes macrocephala koidz
91	13.98	231.1378	213.1271; 195.1166; 185.1323; 171.0797	C_15_H_18_O_2_	Atractylenolide I [21]	0.43	Atractylodes macrocephala koidz
92	14.33	231.1378	213.1272; 185.1325; 157.1011; 143.0855; 129.0694	C_15_H_18_O_2_	Dehydrocostus lactone [30]	0.43	Radix aucklandiae
93	14.33	231.1378	213.1272; 195.1168; 185.1325; 155.0932; 141.0698; 128.0622	C_15_H_18_O_2_	Linderenol [29]	0.87	Radix linderae
94	14.57	279.1215	261.2212; 243.2105; 173.1323; 109.1014; 91.0547	C_15_H_18_O_5_	Aconitine A [29]	4.3	Radix linderae
95	14.57	279.2315	261.2212	C_18_H_30_O_2_	*γ*-Linolenic acid [14]	1.07	Rhizoma drynariae
96	14.72	301.0704	286.1941	C_16_H_12_O_6_	Baicalein II [28]	1.00	Radix scutellariae
97	15.27	381.2045	335.2005; 191.1065	C_24_H_28_O_4_	Senkyunolide P [19]	3.93	Angelica sinensis
98	15.44	191.1065	173.0960; 145.1011; 117.0700; 105.0701; 91.0546	C_12_H_14_O_2_	Z-Ligustilide [19]	0.52	Angelica sinensis
99	15.45	381.2048	363.1941; 335.1997; 293.1533	C_24_H_28_O_5_	Eugelatinolide A [19]	3.15	Angelica sinensis
100	15.82	261.1120	243.2104; 215.1794; 173.1325; 145.1012; 129.0702; 105.0703; 91.0545	C_15_H_16_O_4_	Isolinderalactone [29]	0.38	Radix linderae
101	15.98	499.3768	121.0649	C_32_H_50_O_4_	Acetyl *β*-lactic acid [35]	2.80	Frankincense
102	16.46	852.5253	572.2840; 540.2577; 512.2659; 354.1691	C_49_H_73_NO_11_	8-Linoleic acid-benzoylmesaconine [13]	0.35	Radix aconiti kusnezoffii
103	16.59	866.5411	586.2986; 526.2831; 494.2518	C_50_H_75_NO_11_	8-Linoleic acid-benzoylaconine [13]	0.23	Radix aconiti kusnezoffii
104	16.62	836.5302	556.2900; 524.2636	C_49_H_73_NO_10_	8-linoleic acid-benzoylhepaconine [13]	0.60	Radix aconiti kusnezoffii
105	16.88	828.5244	572.2844; 540.2590; 512.2673	C_47_H_73_NO_11_	8-palmitic acid-benzoylmesaconine [13]	1.45	Radix aconiti kusnezoffii
106	16.93	365.2678	347.2577; 305.2469; 287.2365; 269.2263	C_22_H_36_O_4_	Incensole oxide acetate [19]	2.19	Frankincense
107	17.04	812.5305	556.2896; 524.2642; 496.2780	C_47_H_73_NO_10_	8-palmitic acid-benzoylhepaconine [13]	0.25	Radix aconiti kusnezoffii
108	17.2	513.3556	495.3528; 453.3362; 435.3258; 407.3304	C_32_H_48_O_5_	3-Acetyl-11-ketone-*β*-mastic acids [19]	3.51	Frankincense
109	17.24	135.0804	105.0701; 91.0546; 79.0548	C_9_H_10_O	Cinnamic alcohol [10]	0	Cinnamomum cassia presl
110	17.26	307.2625	289.2519; 271.2418; 215.1787; 201.1635	C_20_H_34_O_2_	Incensole [19]	1.95	Frankincense
111	17.26	307.1172	289.2519; 221.1909; 205.1953; 177.1638	C_16_H_18_O_6_	Cimifugin [25]	1.30	Saposhnikovia divaricata
112	18.10	581.1868	563.3374; 545.3251	C_27_H_32_O_14_	Naringin [32, 33]	−0.52	Qingpi
113	18.43	497.3615	437.3409; 409.3513; 391.3358	C_32_H_48_O_4_	3*α*-acetyl-9,11Deoxidizing-*β*-boswellic acid [19]	2.01	Frankincense
114	18.94	457.3673	421.3467; 407.2956	C_30_H_48_O_3_	*β*-Boswellic acid [35]	0.66	Frankincense
115	20.76	273.2571	229.4793; 217.1954; 163.1483; 149.1324	C_20_H_32_	Cupressene [19]	2.20	Frankincense
116	22.47	152.1064	133.9745; 116.9721	C_9_H_13_NO	Norephedrine [16]	3.94	Ephedra
117	22.65	152.1075	133.9798; 116.9721	C_9_H_14_NO	Pseudodesmethylephedrine [16]	−3.29	Ephedra

**Table 3:** The identified compounds absorbed into blood in DHLP by UPLC-Q-exactive-orbitrap-MS.

No.	Ion form	*t *_*R*_ (min)	Measured value	MS/MS	Formula	Identification	Error (ppm)	Type	Form (prototypes/metabolites)
1	[M + H]^+^	1.08	175.1185	158.0920, 130.0972, 116.0705	C_6_H_14_N_4_O_2_	Arginine	−2.28	Amino acids	Prototypes
2	[M − H]^−^	1.22	191.0191	129.0181, 111.0075	C_6_H_8_O_7_	Citric acid	−3.14	Carboxylic acids	Prototypes
3	[M − H]^−^	1.49	191.0555	173.0081, 121.6582, 111.0075	C_7_H_12_O_6_	Quinic acid	−3.14	Phenols	Prototypes
4	[M + H]^+^	2.38	127.0389	109.0285	C_6_H_6_O_3_	5-Hydroxymethyl-2-furaldehyde	−1.57	Furans	Metabolites
5	[M + H]^+^	5.03	148.1118	133.0885	C_10_H_13_N	5,6,7,8-Tetrahydro-4-methylquinoline	−2.03	Alkaloids	Metabolites
6	[M − H]^−^	5.41	353.0876	191.0345	C_16_H_18_O_9_	Chlorogenic acid	−0.57	Carboxylic acids	Prototypes
7	[M + H]^+^	5.94	417.1172	399.1086, 381.0962, 351.0851	C_21_H_20_O_9_	Puerarin	−1.92	Flavonoids	Prototypes
8	[M − H]^−^	6.99	549.1628	255.0664, 135.0077	C_26_H_30_O_13_	Celery glycyrrhizin	2.73	Flavonoids	Prototypes
9	[M − H]^−^	7.07	417.119	255.0662, 135.0076	C_21_H_22_O_9_	Liquiritin	−0.24	Flavonoids	Prototypes
10	[M − H]^−^	7.38	579.1721	459.1163, 271.0614, 151.0027	C_27_H_32_O_14_	Naringin	0.35	Glycosides	Prototypes
11	[M + H]^+^	7.5	453.1744	291.1217, 273.1113, 259.0956, 243.0646, 219.0645, 205.0493	C_22_H_28_O_10_	5-O-Methylvisammioside	−2.43	Glycosides	Prototypes
12	[M + H]^+^	7.51	319.1147	301.1055, 283.0996, 255.1003, 227.1056, 164.0462	C_17_H_18_O_7_	Aquilarone B	−9.09	Chromones	Prototypes
13	[M − H]^−^	7.7	609.1824	301.0718	C_28_H_34_O_15_	Hesperidin	−0.16	Glycosides	Prototypes
14	[M − H]^−^	7.74	289.0713	245.1544	C_15_H_14_O_6_	Catechin	−1.73	Flavonoids	Prototypes
15	[M + H]^+^	8.28	271.0596	253.1943, 243.2106	C_15_H_10_O_5_	7,8,4′-Trihydroxyisoflavone	−1.84	Flavonoids	Metabolites
16	[M + H]^+^	8.28	271.0596	253.0493, 243.0549	C_15_H_10_O_5_	7,8,4′-Trihydroxyisoflavone	−1.84	Flavonoids	Metabolites
17	[M + H]^+^	8.56	255.0645	237.0537, 227.0698, 199.0749	C_15_H_10_O_4_	Daidzein	−2.74	Flavonoids	Prototypes
18	[M − H]^−^	8.57	253.0506	225.0547	C_15_H_10_O_4_	Chrysophanic acid	0.40	Quinones	Prototypes
19	[M + H]^+^	8.89	461.1069	285.0749, 270.0515	C_22_H_20_O_11_	Wogonoside	−1.95	Flavonoids	Prototypes
20	[M + H]^+^	9.5	271.0596	253.0493, 241.0493, 225.0544, 197.0956, 169.0646	C_15_H_10_O_5_	Baicalein	−1.84	Flavonoids	Prototypes
21	[M + H]^+^	9.82	285.0748	270.0520, 267.0643, 257.0804	C_16_H_12_O_5_	3′-Methoxyldaidzein	−3.16	Flavonoids	Metabolites
22	[M + H]^+^	10.08	233.1523	215.1419, 187.1477, 159.1165, 145.1009, 131.0854	C_15_H_20_O_2_	Costunolide	−5.58	Terpenes	Prototypes
23	[M − H]^−^	10.47	821.3971	351.0568	C_42_H_62_O_16_	Glycyrrhizic acid	0.73	Saponosides	Prototypes
24	[M − H]^−^	11.42	283.0247	239.0346, 183.0443	C_15_H_8_O_6_	Rheinic acid	−0.35	Quinones	Prototypes
25	[M + H]^+^	11.44	285.0748	270.0514	C_16_H_12_O_5_	Wogonin	−3.16	Flavonoids	Prototypes
26	[M − H]^−^	12.44	371.1132	193.0500, 177.0916	C_20_H_20_O_7_	Glycosmisic acid	−1.08	Phenylpropanoids	Prototypes
27	[M + H]^+^	13.97	225.1118	207.1012, 151.0389	C_12_H_16_O_4_	Chuanxionglide II	−1.33	Ester	Prototypes

**Table 4:** The information of 15 key targets.

Gene name	Target	UniProt ID	Degree
AKT1	Serine/threonine-protein kinase AKT	P31749	52
STAT3	Signal transducer and activator of transcription 3	P40763	50
MAPK1	c-Jun N-terminal kinase 3	P53779	50
VEGFA	Vascular endothelial growth factor A	P15692	48
MAPK8	c-Jun N-terminal kinase 1	P45983	45
EGFR	Epidermal growth factor receptor ErbB1	P00533	44
TNF	TNF-alpha	P01375	43
SRC	Tyrosine-protein kinase SRC	P12931	38
HRAS	Transforming protein p21/H-Ras-1	P01112	36
CTNNB1	Axin1/beta-catenin	P35222	34
JUN	Proto-oncogene c-JUN	P05412	33
APP	Beta-amyloid A4 protein	P05067	33
PTGS2	Cyclooxygenase-2	P35354	31
RELA	Nuclear factor NF-kappa-B p65 subunit	Q04206	30
IL2	Interleukin-2	P60568	29
TLR4	Toll-like receptor 4 (by homology)	O00206	28
IL1B	Interleukin-1 beta	P01584	28
CASP3	Caspase-3	P42574	24

## Data Availability

The data used to support the findings of this study are included within the article.
